# Role of dental training and distance of the observer on the perception of apically shifted gingival margin with increased vertical tooth size in the esthetic zone

**DOI:** 10.1002/cre2.692

**Published:** 2022-11-22

**Authors:** Marco Montevecchi, Fabio Paolo Desimini, Nicola Sforza, Simone Bagattoni, Gabriela Piana

**Affiliations:** ^1^ Dental Clinic, Department of Biomedical and Neuromotor Sciences Alma Mater Studiorum—University of Bologna Bologna Italy; ^2^ Private Practice Bologna Italy; ^3^ Private Practice COS Centro Odontoiatrico Sforza Bologna Italy

**Keywords:** esthetics, gingival recession, perception, smiling

## Abstract

**Objectives:**

To evaluate the influence of the gingival contour on the smile esthetics. The influence of size, symmetry, teeth involved in apically shifted gingival margins, and the distance and clinical training of the observer were investigated.

**Materials and Methods:**

Two groups were identified: 33 first‐year dental students (inexperienced) and 40 last‐year students (trained). Each observer expressed four evaluations on four different images assigning a score from 0 to 10. Using a picture of an “ideal” female smile, 10 variants were virtually created by shifting (2 and 4 mm) the gingival contour apically at different sites of the upper incisors and canines. A total of 292 evaluations were collected.

**Results:**

Considering a score >6 for a “pleasant smile,” only one 4 mm single alteration at the canine gingival contour obtained an insufficient score. “Observational distance” and “clinical training” did not influence the final score, while size and symmetry of alterations displayed a significant role.

**Conclusions:**

The dental training of the observer and a close interpersonal distance seemed to be irrelevant in the esthetic perception of gingival margin alterations.

## Clinical significance

Gingival contour seems to play a marginal role on the smile esthetics. The dental training of the observer appears irrelevant.

## 1 INTRODUCTION

The smile may be conceived as an articulated dynamic of the whole face, and the proportion between different anatomical parts creates that uniqueness that each individual represents. It is therefore undoubtedly complex to objectively identify clear canons that describe an ideal smile. The current esthetic requirements have led to a scientific deepening of the topic, recognizing three fundamental units of the perioral district: lips, teeth, and periodontal tissues (Bhuvaneswaran, 2010; Garber & Salama, 1996). These units are connected through lines of symmetry, proportions, and colors (Bukhary et al., 2007; Calamia & Wolff, 2015; David et al., 2015; Manipal et al., 2014). In this articulated balance, the anatomy of the gingival margins is crucial (Giancotti et al., 2011; Spear et al., 2006). The gingival margin follows and reflects the underlying bone architecture, and its features are influenced by several factors (Chu et al., 2009; Seixas et al., 2012) such as tooth position, contact point, periodontal phenotype, dental morphology, and cementoenamel junction (CEJ).

A harmonic architecture of the smile follows some well‐established esthetic rules: the gingival margin of the central incisor has to be similar to that of the canine, slightly apical to that of the lateral incisor (0.5−2.0 mm); interproximally there must be a pyramidal gingival papilla, which correctly fills the interdental space. Gingival asymmetry is considered a disturbing factor: the closer to the midline, the stronger the impairment of the smile esthetics (Kokich et al., 1999). When the smile esthetics is altered, limitations in social relationships can be suffered (da Cunha et al., 2017). In some people, periodontal disorders can limit their smile confidence and reduce their communication skills, leading to social isolation. Among the periodontal disorders, gingival recessions play a major role in smile esthetics (Lindhe & Lang, 2015; Löe et al., 1992). These periodontal lesions can have different causes (Van Dyke, 2008; Jati et al., 2016; Jorgensen & Nowzari, 2001) and become esthetically evident when they are present in individuals with large gingival display, such as gummy smile (Khan & Abbas, 2014; Nart et al., 2014; Robbins, 1999; Waldrop, 2008).

From an epidemiological point of view, periodontal recessions are a widespread alteration (Albandar & Kingman, 1999; Marini et al., 2004; Mythri et al., 2015): 50% of people between 18 and 64 and 88% of people aged 65 and older have one or more sites showing gingival recession (Kassab & Cohen, 2003). Despite the high prevalence, however, patients are frequently unaware of their presence (Nieri et al., 2013; Saffarpour et al., 2016).

The latter aspect raises some doubts about the concrete esthetic relevance of gingival recession. This aspect has not been studied in depth and, as a paradox, while the parameters of an ideal gingival contour are quite well defined, a clear esthetic relevance of recessions is still unknown.

The present study aims to investigate the esthetic role of an apically shifted gingival contour involving maxillary anterior teeth. Furthermore, we investigated whether the dental training and the observation distance of the observer may influence esthetic judgment.

## 2 MATERIALS AND METHODS

**Figure 1:**
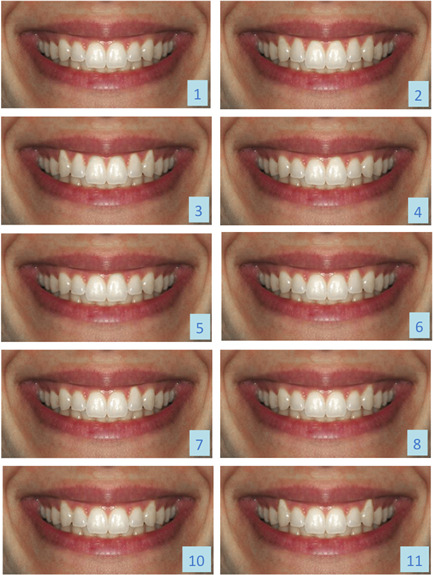
Ten of the 11 smile variants used during the study. Number 1, control picture; 2, 2 mm frontal group recessions; 3, 4 mm frontal group recessions; 4, 2 mm unilateral central incisor recession; 5, 4 mm unilateral central incisor recession; 6, 2 mm unilateral incisor recession; 7, 4 mm unilateral lateral incisor recession; 8, 2 mm unilateral canine recession; 10, 2 mm bilateral canine recessions; 11, 4 mm bilateral canine recessions.

**Figure 2:**
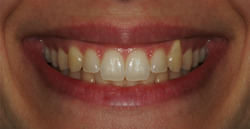
Variant number 9, 4 mm unilateral canine recession

A 20‐year‐old Caucasian female was selected for her harmonious smile. Lips, teeth, and gingiva were healthy with an ideal anatomical architecture. Starting from a front view picture of the smile, referred to as the control picture, 10 variants were created by apically shifting the gingival contour in different sites (Figures 1 and 2): for each tooth, either a 2 or a 4 mm gingival margin transposition was created. A uni‐ and a bilateral‐variant of gingival margin alteration was created for canines only. A specific software was used for the graphic elaboration (Adobe Photoshop CC 2012—Adobe Systems Software Ireland Ltd; headquarters: Adobe Systems Incorporated 345 Park Avenue). Each obtained image was then printed on photographic paper with a 1:1 dimension, corresponding to the real face proportions. Eleven pictures, the control picture, and the 10 variants, were included in the protocol.

Two groups of examiners were selected among the students at the School of Dentistry, Bologna University, Bologna, Italy: the first, named *“inexpert group,”* was composed of 33 first‐year students (21 males and 12 females); the second, named *“trained group,”* included 40 last year students (26 males and 14 females). The two groups resulted balanced for gender (female percentage: 36% and 35%, respectively).

Each observer gave four evaluations using a visual analogue scale on two different images observed separately at two predefined distances, starting from 120 cm and concluding at 45 cm. Each image was displayed for 30 s. A time lap of 15 min was waited between the evaluations to discard short‐term memory (Baddeley et al., 1975; Cowan, 2008). For both groups, images were randomized using a random number generator (https://it.piliapp.com/random/number/) until reaching at least two observations per image at both distances. The value assigned ranged from 0 to 10, considering 0 as “unacceptable,” 10 as “ideal,” and 6 as the minimum value of appreciation. The average score for each image was calculated.

### 2.1 Statistical analysis

The null hypothesis states that the dental training and the observation distance of the observer do not influence the perceptual outcome. The statistical analysis was carried out considering the arithmetic mean and standard deviation of the scores expressed by the two groups at a distance of 45 and 120 cm. The *t*‐test was used to evaluate the statistical significance of the difference between the scores. To evaluate the effect of training, distance, and type of alteration on the student's assessment, a multilevel analysis was performed with a mixed effects model at three levels: alteration type, training, and distance. The unit of analysis was the alteration type since it was not possible to match each student with the scores awarded in the four voting sessions. The significance level *α* was set to 0.05.

## 3 RESULTS

A total of 292 evaluations were collected. For each image, the average score assigned is summarized in Table 1. The smile with a 2 mm gingival recession on the frontal elements (six frontal teeth) obtained the highest score among all variants, followed by the control picture and the 2−4 mm gingival recessions on central incisors. Considering a score of 6 as the limit for a pleasant smile, only the 4 mm alteration on a single canine obtained an insufficient average score (5.62).

**Table 1:** Average scores of the evaluations expressed for each image

Anatomical variants *N*°	Description	Alteration (mm)	Score	SD
1	Control picture	/	7.54	0.86
2	Front elements	2	7.80	1.19
3	Front elements	4	6.50	1.17
4	Central incisor	2	7.32	1.16
5	Central incisor	4	7.35	1.13
6	Lateral incisor	2	7.00	1.00
7	Lateral incisor	4	6.32	1.55
8	Unilateral canine	2	7.16	1.21
9	Unilateral canine	4	5.62	1.85
10	Bilateral canines	2	7.21	1.44
11	Bilateral canines	4	6.93	1.00

*Note*: Anatomical variants are named with the number assigned during the study and synthetically described.

Abbreviation: SD, standard deviation.

Comparing each score with that obtained for the unilateral 4 mm canine alteration, a significant difference was detected for the control picture (*p* = .01), the 2 mm front elements alteration (*p* = .01), and the 2−4 mm central incisor alteration (*p* = .04 both).

The evaluations expressed by the two groups at 45 and 120 cm distances respectively are summarized in Table 2. For both groups, distance did not significantly influence the evaluation score.

**Table 2:** Average scores assigned by inexpert group and trained group to the anatomical variants at the two study distances.

	45 cm distance				120 cm distance			
Anatomical variants N°	Inexpert		Trained		Inexpert		Trained	
	Score	SD	Score	SD	Score	SD	Score	SD
1	7.66	0.47	6.83	0.69	8.12	0.37	7.33	0.94
2	7.5	1.12	7.66	0.75	8.71	1.26	7.16	0.69
3	6.66	1.37	6.66	1.25	6.50	0.50	6.66	1.25
4	7.66	0.75	7	1.29	7.57	0.90	8	1.15
5	7.16	1.21	7.83	0.37	7.25	0.75	7.16	1.21
6	7	0.58	6.83	1.07	6.87	1.70	7	1.15
7	6.33	1.11	6.50	0.96	5.66	1.80	6.74	1.75
8	7.16	1.07	6.66	1.25	7.71	0.76	7	1.29
9	5.33	1.11	6	1.73	5.14	1.97	6.14	1.81
10	7.16	1.77	7.33	1.49	7.5	0.96	7	1.26
11	6.50	1.38	6.33	0.47	7.22	1.11	7.37	1.41

Abbreviation: SD, standard deviation.

Table 3 shows the multilevel analysis with a mixed‐effect model to assess the effect of dental training, distance, and type of alteration on the observer's judgment. The proxemic distance as the training of the observer did not influence the final evaluation.

**Table 3:** Multilevel analysis with mixed effects model

Parameter	Score coefficient	*p*	95% CI
Intercept	7.092	.000	6.552/7.631
Training	0.188	.230	−0.120/0.496
Distance	0.036	.819	−0.271/0.342
Image: 4 mm unilateral canine	−1.557	.003	−2.267/−0.847
Image: 2 mm bilateral canines	−0.886	.016	−1.604/−0.169

*Note*: Influence of training, distance, and type of alteration on the observer's evaluation.

Abbreviation: CI, confidence interval.

## 4 DISCUSSION

The present study investigated the role of gingival contour in the perceived smile harmony when evaluated by an external observer in a frontal view. The observation distance, the dental training of the observer, and the size and/or symmetry of gingival alterations were considered.

From the results obtained, a slight apical shifting of the gingival margin localized on the maxillary anterior teeth seemed to have no influence on the esthetic perception of the observer.

Only a 4 mm unilateral canine alteration was responsible for a relevant worsening of the esthetic evaluation.

The most appreciated pictures, the control picture, and the front group alterations, were characterized by a symmetrical contour of the gingival margin. The third most appreciated pictures were the 2−4 mm gingival recessions on central incisors. This finding suggests that symmetry and proximity to the midline are able to reduce the esthetic impact of gingival contour alterations. In a previous clinical study, Kokich et al. (1999) highlighted that the closer the gingival asymmetry to the midline, the worse the esthetic perceived impairment. This conclusion contrasts with the present findings. Certainly, the relation between symmetry and esthetics is not strictly linear. First of all, the training of an observer can play a role. Even if the present study failed to identify this role, it has been reported that inexperienced observers frequently are unable to recognize certain asymmetrical dental alterations, considering a smile pleasant despite the presence of anatomical discrepancy (Kokich et al., 2006). The results of a recent investigation reinforced the controversial role of symmetry. In this paper, a slightly crooked smile was surprisingly judged more attractive than a symmetrical one (Helwig et al., 2017). In the present study, the symmetry of the defects was not investigated for the central and lateral incisors. Supported by the lower prevalence of such recessions compared to canines (Albandar & Kingman, 1999; Jati et al., 2016), this choice was taken a priori to reduce the number of images included. In line with previous studies, the observer's dental training did not affect the esthetic perception (Cotrim et al., 2015; Kokich et al., 1999). However, the literature is still not concordant in this aspect. In recent studies by Saffarpour et al. (2016) and by Correa et al. (2013), a perceptive difference between laypeople and experienced professionals concerning esthetics was identified. It was interesting to observe that in the study by Correa et al., the “expert” orthodontists perceived a discrepancy in the gingival margin symmetry between the maxillary canines starting from 1 mm, while the inexperienced began to notice it from 2 mm. In the present study, almost all the alterations did not obtain a significantly worse judgment when compared to the control picture. It could be speculated that students are trained but inexperienced observers, more similar to ordinary people than experienced dental professionals. This consideration helps to explain why only the alterations exceeding 2 mm from the ideal gingival contour have generally led to a reduction in the assigned score.

The present study evaluated the role of the observer's distance, identifying two different distances referring to Edward Hall's proxemic theories (Aiello & De Carlo Aiello, 1974; Hall, 1968). This variable did not influence the judgment. This result suggests that even at limited distances, closer to the sphere of an “intimate relationship,” observers may not be able to identify gingival disharmonies.

Another interesting aspect influencing the role of gingival recessions in smile esthetics is the degree of dento‐gingival display while smiling. A young smile commonly displays about 1−4 mm of gingiva apically to the CEJ and gradually reduces during aging (Dickens et al., 2002). This consideration implies that even a large gingival recession has a potentially very narrow space to produce a negative influence on smile esthetics. Another consideration is the color of the exposed root surface. In the present study, during the software processing, the color of the tooth crown was adopted to simulate the root exposure. In a clinical condition, the exposed root may have a clearly different color from the crown due to extrinsic and intrinsic pigmentations. This variable may be relevant for the esthetic perception of the observer and for this reason it is not correct to make a direct comparison to gingival recession. In fact, the alterations created for the present study are more similar to a surgical crown lengthening followed by a tooth restoration rather than a gingival recession. It can be concluded that the present findings can give thought to just a hint of the possible esthetic role of gingival recession. Thus, future investigations considering this aspect are suggested.

Inside each group, the distribution of gender resulted nonhomogeneous due to a prevalence of males, even if a similar proportion of females is observed. Thus, it was not possible to investigate the role of this variable that could have been potentially relevant (Geron & Atalia, 2005).

In conclusion, gingival contour apical shifting seems to play an overall marginal role in the esthetic perception of a smiling front view and is not affected by the observer's proximity. Controversial is the role of marginal alteration asymmetry that could be a relevant aspect to claim for further investigations.

## AUTHOR CONTRIBUTIONS

Dr. Montevecchi Marco has made substantial contributions to the conception, design of the study, and interpretation of data. He has been involved in drafting the manuscript and revising it critically. Dr. Fabio Paolo Desimini has made substantial contributions to the acquisition, analysis, and interpretation of data. He has also been involved in drafting the manuscript. Dr. Nicola Sforza has made substantial contributions to the conception of the study design and has been involved in drafting the manuscript. Dr. Simone Bagattoni has been involved in data acquisition and revising the manuscript for important intellectual content. Prof Gabriela Piana has been involved in revising the manuscript critically for important intellectual content. All authors have given final approval of the version to be published and have agreed to be accountable for all aspects of the work in ensuring that questions related to the accuracy or integrity of any part of the work are appropriately investigated and resolved.

## CONFLICT OF INTEREST

The authors declare no conflict of interest.

## Data Availability

The data that support the findings of this study are available from the corresponding author upon reasonable request.
